# Evaluation of radionuclides and decay simulation in a terrestrial environment for health risk assessment

**DOI:** 10.1038/s41598-017-16659-w

**Published:** 2017-11-28

**Authors:** I. N. Doyi, D. K. Essumang, S. B. Dampare, D. Duah, A. F. Ahwireng

**Affiliations:** 1 National Radioactive Waste Management Centre, Ghana Atomic Energy Commission, P. O., Box LG 80 Legon-Accra, Ghana; 2 Department of Chemistry, School of Physical Sciences, University of Cape Coast, Cape Coast, Ghana; 3 Graduate School of Nuclear & Allied Sciences, Ghana Atomic Energy Commission, P. O. Box AE 1 Kwabenya-Accra, Ghana; 4 Department of Environmental Sciences, Faculty of Science and Engineering, Macquarie University, Sydney, NSW 2109 Australia

## Abstract

This study is to assess the natural radioactivity level in soil samples in communities bordering the Tano Basin in Ghana. The radioactivity concentration of ^238^U, ^232^Th and ^40^K have been determined using γ-ray spectrometry, moreover, the absorbed dose rates and annual effective dose were calculated. MATLAB R2013 script was written to simulate the decay of the radionuclides ^238^U, ^232^Th and ^40^K using their respective half-lives. This is to determine the future impact of natural radionuclides and estimate future anthropogenic inputs. The level ^238^U, ^232^Th, and ^40^K ranged from (1.60 to 21.3), (2.78 to 32.2) and (111 to 528) with average values of be 8.65 Bqkg^−1^, 12.5 Bqkg^−1^ and 214 Bqkg^−1^ respectively in soil. The activity concentrations were lower than United Nations Scientific Committee on the Effects of Atomic Radiation guidelines for ^238^U, ^232^Th and ^40^K. The absorbed dose rates and annual effective dose were found to be in range of 7.79 to 37.8 nGy h^−1^ and 9.56E + 00 to 4.64E + 01 µSvy^−1^ respectively. The overall annual effective dose was lower than the allowable limit of 1mSvy^−1^ set by International Commission on Radiological Protection^.^ H_ex_, H_in_ and excess lifetime cancer risk (ELCR) were calculated and found to be within internationally recommended values.

## Introduction

Soil is not only a source of continuous radiation exposure to humans^1^, it is also a medium of migration and transfer of radionuclides to biological systems^1,2^. Subsequently, soil can provide an indication of anthropogenic radiological contamination in the environment^1,3^. Soil radioactivity is also affected by man-made activities^4^. The evaluation of the radioactive components in soil is critical in understanding the behavior of radioactivity in the ecosystem, due to its impact on the total absorbed dose via ingestion, inhalation and external irradiation^5,6^. Yet, soil radioactivity studies largely focus on radiation protection and establishing baseline data for future radiation impact assessments^7^. They also estimate changes in environmental radioactivity caused by nuclear, industrial, and other human activities^8^.

Natural radioactivity arises mainly from primordial radionuclides, such as potassium-40 (^40^K) and the radionuclides from uranium-238 (^238^U), thorium-232 (^232^Th) series and their decay products, which are present at trace levels in all ground formations^9^. The amount of radioactivity in soil varies widely and significantly affects gamma radiation levels, which in turn can be used for the assessment of terrestrial gamma dose rates^10,11^. Natural radiation is the main source of radiation exposure in humans^12^ and has led to studies of radiation levels, doses from natural radiation sources and its effects on health. Further, studying the distribution of radionuclides in the environment improves our understanding of radiation damage, and, therefore, is of great importance as a reference when standards and regulatory control actions on radiation protection are established^5,13,14^.

The twelfth United Nations Development Programme Sustainable Development Goal (SDG) aims to achieve responsible consumption and production^15^. The SDG identifies proper disposal of toxic wastes and pollutants as a critical priority in achieving this overarching goal^15^. For instance, the disposal of toxic wastes from oil and gas drilling activities that contain radionuclides and trace metals should be an important target in achieving this goal in oil producing countries. This is important as radionuclides in the soil can be leached into and transported via groundwater, drainage, and dust, and incorporated into the food chain^16–18^.

The radiological impact of oil and gas activities in the production land areas of Delta State, Nigeria has indicated that soil radioactivity levels for Otorogu, Ughelli West, Afiesere and Uzere West and East and the host communities of Olomoro, Uzere, and Emeragha have exceeded the maximum recommended value of 1mSvy^−1^ as set by ICRP^19^ for the public and non-nuclear industrial environment. This is an indication that the environment in these host communities has been impacted radiologically due to the local oil and gas activities. Despite the lack of evidence for public radiation exposure in the soil as a consequence of oil and gas activities in the communities along Tano Basin, Ghana, the Nigerian example is an eye-opener. The communities along the Tano Basin are mostly rural. There was flaring of natural gas and the only guideline regulating the discharge of produced water into the open sea at the Tano Basin is the Environmental Protection Agency’s guideline of oil-in-water content of 29 mg/L^20^. These significant environmental concerns are capable of negatively impacting agricultural activities in these communities and threatening food security^21^ in the near future. Some of the communities are also host to gas pipelines that are linked to a gas processing factory located in nearby Atuabo. Background radioactivity data will be collated as a future indicator for how well oil and gas wastes are being managed in order to achieve SDG goal 12.

This study provides free soil radioactivity testing to the lower-income population of Ghana to assist sustainable backyard gardening and provides peace of mind when consuming backyard garden produce. Residents are provided feedback on soil contamination levels and are advised on how to reduce radiation exposure. Hence the main objectives of this study are: (1) to develop a modelling tool that will be used to predict radionuclide levels; (2) to evaluate the potential for radiation exposure and the health risks to the public associated with estimated doses; and (3) to establish background data on naturally occurring radioactive material (NORM) contamination for future referencing as a result of oil and gas drilling in Ghana. Finally, this study intends to attract the attention of governments in developing countries to integrate environmental sustainability into their developmental policies for the rapid attainment of SDG 12.

## Results

### Activity concentrations

The levels of ^238^U, ^232^Th, ^40^K in soil samples collected from communities along Tano Basin in Ghana are summarised in Table 1. ^238^U, ^232^Th, and ^40^K ranged from 1.60–21.3 Bqkg^−1^, 2.78–32.2 Bqkg^−1^ and 111–528 Bqkg^−1^ respectively with mean values of 8.65 Bqkg^−1^ (^238^U), 12.5 Bqkg^−1^(^232^Th) and 214 Bqkg^−1^ (^40^K).

**Table 1:** Radioactivity intensities (Bqkg^−1^) of radionuclides. Values displayed to 3 significant figures. Plus-minus values represent the instrument precision.

Sample	Activity Concentration		
	^238^U	^232^Th	^40^K
SS1	8.96 ± 1.5	9.12 ± 1.2	298 ± 44.7
SS2	6.25 ± 0.4	19.6 ± 1.6	226 ± 34.0
SS3	9.32 ± 1.4	10.1 ± 1.5	302 ± 45.2
SS4	4.15 ± 0.6	6.22 ± 0.9	132 ± 18.8
SS5	12.9 ± 1.1	8.06 ± 1.5	353 ± 52.9
SS6	1.60 ± 0.4	3.09 ± 0.3	124 + 1.50
SS7	14.5 ± 2.2	16.3 ± 2.5	263 ± 39.2
SS8	12.7 ± 1.9	8.13 ± 1.2	134 ± 20.1
SS9	13.4 ± 5.4	29.8 ± 2.8	170 ± 25.5
SS10	3.24 ± 0.2	3.01 ± 0.2	127 ± 1.30
SS11	21.3 ± 1.4	26.0 ± 1.0	147 ± 22.0
SS12	5.92 ± 0.8	15.2 ± 1.9	142 ± 4.20
SS13	7.90 ± 0.4	3.81 ± 0.4	129 ± 1.30
SS14	5.20 ± 0.8	7.43 ± 1.1	189 ± 28.1
SS15	8.96 ± 2.0	32.2 ± 1.8	340 ± 51.0
SS16	4.62 ± 0.3	3.84 ± 0.3	129 ± 61.4
SS17	9.26 ± 2.3	19.2 ± 1.2	232 ± 34.8
SS18	8.87 ± 1.1	17.1 ± 2.4	354 ± 13.9
SS19	4.56 ± 0.6	20.3 ± 1.6	273 ± 7.40
SS20	13.9 ± 0.2	12.91 ± 0.3	136 ± 0.40
SS21	10.7 ± 0.2	17.7 ± 0.9	213 ± 8.70
SS22	8.36 ± 0.8	6.55 ± 0.8	174 ± 3.50
SS23	5.59 ± 0.8	2.78 ± 0.4	528 ± 79.1
SS24	6.08 ± 0.9	9.27 ± 1.4	111 ± 1.50
SS25	9.05 ± 2.3	6.01 ± 2.0	205 ± 30.7
SS26	7.45 ± 0.4	11.4 ± 1.5	136 ± 1.50
Range	**1.60**–**21.3**	**2.78**–**32.2**	**111**–**528**
Mean	**8.65**	**12.5**	**214**

### Absorbed dose rate, annual effective dose and radiological risk assessment due to radioactivity in soil samples

The estimated absorbed dose rates and annual effective dose rates of samples are shown in Table 2. The absorbed dose rate ranged from 7.79 ± 0.3 and 37.8 ± 5.2 nGyh^−1^ with a mean value of 20.5 ± 2.3 nGyh^−1^. The mean annual effective dose for the communities was 2.51E + 01 ± 2.9E + 00 µSvy^−1^ calculated using equation 4. The calculated radiation hazards of radium equivalent activity (Ra_eq_), excess lifetime cancer risk (ELCR), external hazard index (H_ex_) and internal hazard index (H_in_) are equally presented in Table 3. The mean values of Ra_eq_, ELCR, H_ex_ and H_in_ are 43.4 Bqkg^−1^, 8.80E-05 ± 1.0E-05, 0.14 ± 0.02, 0.12 ± 0.01 respectively.

**Table 2:** Estimated absorbed dose rate (*D*) and annual effective dose (*E*) and the percentage contribution of each radionuclide ^238^U, ^232^Th and ^40^K.

Sample	Absorbed Dose, nGyh^−1^	Annual Effective Dose, µSvy^−1^	Percentage contribution of radionuclides to absorbed dose rate, %		
			^238^U	^232^Th	^40^K
SS1	22.1 ± 3.3	2.71E + 01 ± 4.0E + 00	18.7	24.9	56.3
SS2	24.2 ± 2.5	2.97E + 01 ± 3.1E + 00	12.0	49.0	39.0
SS3	22.9 ± 3.5	2.82E + 01 ± 4.2E + 00	18.7	26.6	54.7
SS4	11.2 ± 1.6	1.37E + 01 ± 2.0E + 00	17.1	33.6	49.3
SS5	25.5 ± 3.6	3.13E + 01 ± 4.4E + 00	23.3	19.1	57.6
SS6	7.8 ± 0.4	9.56E + 00 ± 5.0E-01	9.49	24.0	66.6
SS7	27.5 ± 4.1	3.38E + 01 ± 5.1E + 00	24.3	35.6	39.9
SS8	16.4 ± 2.5	2.01E + 01 ± 3.0E + 00	35.9	30.0	34.2
SS9	31.2 ± 5.2	3.83E + 01 ± 6.4E + 00	19.8	57.5	22.7
SS10	8.6 ± 0.3	1.06E + 01 ± 3.6E-01	17.4	21.1	61.6
SS11	31.7 ± 2.2	3.89E + 01 ± 2.7E + 00	31.1	49.6	19.3
SS12	17.8 ± 1.7	2.19E + 01 ± 2.1E + 00	15.3	51.4	33.3
SS13	11.3 ± 0.4	1.39E + 01 ± 5.3E-01	32.2	20.3	47.5
SS14	14.8 ± 2.2	1.81E + 01 ± 2.7E + 00	16.3	30.4	53.4
SS15	37.8 ± 4.2	4.64E + 01 ± 5.1E + 00	11.0	51.5	37.5
SS16	9.8 ± 2.9	1.21E + 01 ± 3.6E + 00	21.7	23.6	54.6
SS17	25.6 ± 3.3	3.14E + 01 ± 4.0E + 00	16.7	45.5	37.8
SS18	29.2 ± 2.6	3.58E + 01 ± 3.2E + 00	14.0	35.4	50.6
SS19	25.8 ± 1.6	3.16E + 01 ± 1.9E + 00	8.18	47.7	44.2
SS20	19.9 ± 0.3	2.45E + 01 ± 3.4E-01	32.3	39.1	28.5
SS21	24.5 ± 1.0	3.01E + 01 ± 1.2E + 00	20.2	43.6	36.3
SS22	15.1 ± 1.0	1.85E + 01 ± 1.2E + 00	25.6	26.3	48.1
SS23	26.3 ± 3.9	3.22E + 01 ± 4.8E + 00	9.83	6.39	83.8
SS24	13.02 ± 1.3	1.60E + 01 ± 1.6E + 00	21.6	43.0	35.4
SS25	16.33 ± 3.5	2.01E + 01 ± 4.3E + 00	25.6	22.2	52.2
SS26	15.9 ± 1.1	1.96E + 01 ± 1.4E + 00	21.6	43.0	35.4
Minimum	**7.8 ± **0.3	**9.56E + 00 ± **3.4E-01	**8.18**	**6.39**	**19.3**
Maximum	**37.8 ± **5.2	**4.64E + 01 ± **6.4E + 00	**35.9**	**57.5**	**83.8**
Mean	**20.5** ± 2.3	**2.51E + 01** ± 2.9E + 00	**20.1**	**34.4**	**45.8**

**Table 3:** Radium equivalent (Ra_eq_), excess lifetime cancer risk (ELCR), the external hazard (H_ex_), and the internal hazard index (H_in_) estimated for the communities.

Sample	Radium Equivalence (Ra_eq_), Bq/kg	ELCR	H_in_	H_ex_
SS1	44.9	9.49E-05 ± 1.4E-05	0.15 ± 0.02	0.12 ± 0.02
SS2	51.7	1.04E-04 ± 1.1E-05	0.16 ± 0.02	0.14 ± 0.01
SS3	47.0	9.87E-05 ± 1.5E-05	0.15 ± 0.02	0.13 ± 0.02
SS4	23.2	4.80E-05 ± 7.0E-06	0.07 ± 0.01	0.06 ± 0.01
SS5	51.6	1.10E-04 ± 1.5E-05	0.17 ± 0.02	0.14 ± 0.02
SS6	15.6	3.35E-05 ± 1.8E-06	0.05 ± 0.00	0.04 ± 0.00
SS7	58.1	1.18E-04 ± 1.8E-05	0.20 ± 0.03	0.17 ± 0.02
SS8	34.7	7.04E-05 ± 1.1E-05	0.13 ± 0.02	0.09 ± 0.01
SS9	67.0	1.34E-04 ± 2.3E-05	0.22 ± 0.05	0.19 ± 0.03
SS10	17.3	3.70E-05 ± 1.3E-06	0.06 ± 0.00	0.05 ± 0.00
SS11	69.8	1.36E-04 ± 9.3E-06	0.25 ± 0.02	0.19 ± 0.01
SS12	38.5	7.66E-05 ± 7.2E-06	0.12 ± 0.01	0.10 ± 0.01
SS13	23.3	4.87E-05 ± 1.9E-06	0.08 ± 0.00	0.06 ± 0.00
SS14	30.4	6.35E-05 ± 9.5E-06	0.10 ± 0.01	0.08 ± 0.01
SS15	81.2	1.62E-04 ± 1.8E-05	0.24 ± 0.03	0.22 ± 0.02
SS16	20.0	4.22E-05 ± 1.2E-05	0.07 ± 0.02	0.05 ± 0.02
SS17	54.6	1.10E-04 ± 1.4E-05	0.17 ± 0.03	0.15 ± 0.02
SS18	60.6	1.25E-04 ± 1.1E-05	0.19 ± 0.02	0.16 ± 0.02
SS19	54.6	1.11E-04 ± 6.7E-06	0.16 ± 0.01	0.15 ± 0.01
SS20	42.9	8.56E-05 ± 1.2E-06	0.15 ± 0.00	0.12 ± 0.00
SS21	52.3	1.05E-04 ± 4.3E-06	0.17 ± 0.01	0.14 ± 0.01
SS22	31.1	6.47E-05 ± 4.2E-06	0.11 ± 0.01	0.08 ± 0.01
SS23	50.2	1.13E-04 ± 1.7E-05	0.15 ± 0.02	0.14 ± 0.02
SS24	27.8	5.59E-05 ± 5.7E-06	0.09 ± 0.01	0.08 ± 0.01
SS25	33.4	7.02E-05 ± 1.5E-05	0.11 ± 0.03	0.09 ± 0.02
SS26	34.1	6.85E-05 ± 4.8E-06	0.11 ± 0.01	0.09 ± 0.01
Minimum	**15.6**	**3.35E-05 ± **1.2E-06	**0.05 ± **0.00	**0.04 ± **0.00
Maximum	**81.2**	**1.62E-04 ± **2.3E-05	**0.25 ± **0.05	**0.22 ± **0.03
Mean	**43.4**	**8.80E-05 ± **1.0E-05	**0.14 ± **0.02	**0.12 ± **0.01

## Discussion

The average levels of ^238^U (8.65 Bqkg^−1^) ^232^Th (12.5 Bqkg^−1^) and ^40^K (214 Bqkg^−1^) are lower than international data of 35, 30 and 400 Bqkg^−1^, respectively published by United Nations Scientific Committee of Effects of Atomic Radiation^9^. Ellonyi has the highest concentration of ^238^U. The highest value of ^232^Th was recorded in Nyale Kplole whilst the highest level of ^40^K was observed in Half-Assini when compared with the concentrations of all the other samples. The reason could be attributed to differences in their geological nature. The activity concentrations of ^238^U, ^232^Th and ^40^K from this study were compared to levels with from Sudan as (18.9–26.5 Bqkg^−1^), (19.1–31.4 Bqkg^−1^), (187.6–385.6 Bqkg^−1^). India recorded (8.89–56.7 Bqkg^−1^), (137.3–334.5 Bqkg^−1^), (823.6–1064.9 Bqkg^−1^) with that from Palestine being (9.7–83.5 Bqkg^−1^), (5.3–44.8 Bqkg^−1^) and (10.2–404 Bqkg^−1^). The levels from Nigeria are (2.87 ± 0.15 to 7.14 ± 0.14 Bqkg^−1^), (1.29 ± 0.02 to 5.53 ± 0.02 Bqkg^−1^), (2.73 ± 0.03 to 66.5 ± 0.81 Bqkg^−1^) respectively^11,22–24^. The levels of radionuclide-specific activity concentrations from the present study are within range of the cited studies from other parts of the globe.

The estimated mean radium equivalent activity of 43.4 Bqkg^−1^ is far lower than the world mean value of 370 Bqkg^−1^ reported by OECD^25^. The International Commission on Radiological Protection (ICRP)^26,27^ has recommended the annual effective dose equivalent limit of 1 mSvy^−1^ for the individual members of the public and 20 mSvy^−1^ for the radiation workers. The total absorbed dose in the study area ranges from 7.79 to 37.8 nGyh^−1^ with an average value of 20.5 nGyh^−1^. The corresponding annual effective doses range from 9.56E + 00 to 4.64E + 01 µSvy^−1^ with an average value of 2.51E + 01 µSvy^−1^ respectively while the worldwide average annual effective dose is approximately 0.5 mSvy^−1^ and the results for individual countries being generally within the 0.30–0.60 mSvy^−1^ range for indoors^9^. The mean annual effective dose of 2.51E + 01 ± 2.9E + 00 µSv/y from this study is one order magnitude less than the worldwide average (0.05 mSv.y^−1^) as reported by UNSCEAR. The calculated values of H_ex_ and H_in_ for the soil samples studied range from 0.04 to 0.22 and 0.05 to 0.25 respectively. Since these values are lower than unity, therefore, according to the Radiation Protection 112^28^ report, soil from these regions is safe and can be used as construction material without posing any significant radiological threat to the population. As a rule, the greater the value of Hazard index above unity, the greater is the level of concern, particularly in cases of the exposure of children. H_ex_ and H_in_ must be less than unity to be considered negligible^29^ as is suggested in the present study. This is confirmed by the Excess Lifetime Cancer Risk that is within the accepted limit of 3.35E-05 to 1.62E-04 with an average of 8.80E-05.

All the radiological risk parameters of Ra_eq_, ELCR, H_in_ and H_ex_ are within accepted regulatory limits. Nevertheless, Ghana being a signatory to the SDGs should adopt international conventions in protecting the poor and vulnerable populace from the ramifications of environmental pollution as part of its efforts in achieving SDG 12. It is incumbent on the Government of Ghana through the Nuclear Regulatory Authority, Ghana to put in place a graded approach and national framework to regulations for the management of NORMs, for the extractive industry in general and for the discharge of produced water specifically. This study adopted the approach of ARPANSA Safety Guide^30^. The ARPANSA Safety Guide takes into consideration recently developed international guidance on NORM management. The drivers of graded approach are based on the premise that regulation will not always be the appropriate approach for dealing with NORM. Country-specific guidelines are therefore needed in this direction.

The activity concentrations of the radionuclides were predicted using the Forward Differential Approach and a written MATLAB R13 script based on their current measured concentrations. Different year considerations were chosen to estimate the extent of decay. From the predicted results, it was observed that there was no significant variance in the predicted activity concentrations from the measured or experimental activity concentrations for ^238^U and ^232^Th whilst the activity concentration of ^40^K showed variation from the measured activity concentrations that are quite significant. From the decay equation

1 $${\boldsymbol{A}}={{\boldsymbol{A}}}_{0}{{\boldsymbol{e}}}^{-{\boldsymbol{\lambda }}t}$$

**Figure 1:**
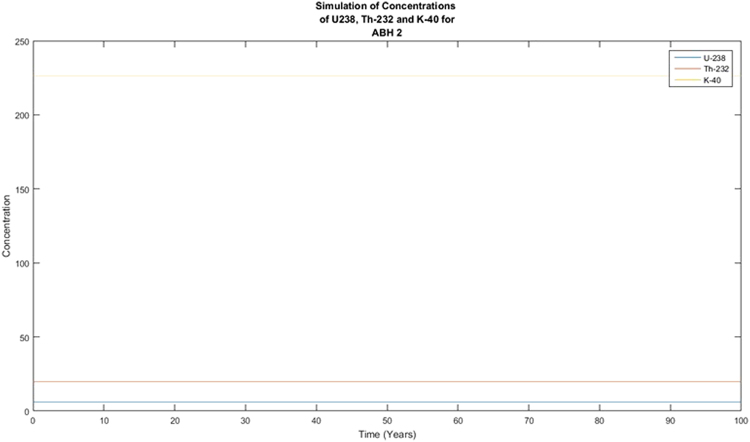
Simulation of the decay of the radionuclides ^238^U, ^232^Th and ^40^K in 100 years.

**Figure 2:**
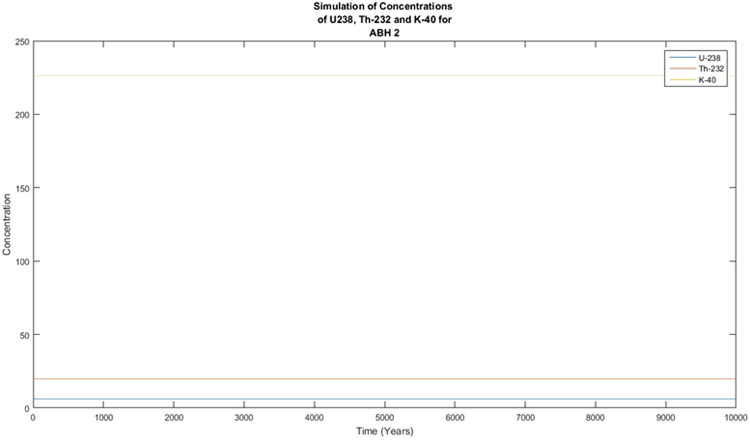
Simulation of the decay of the radionuclides ^238^U, ^232^Th and ^40^K in 10000 years.

**Figure 3:**
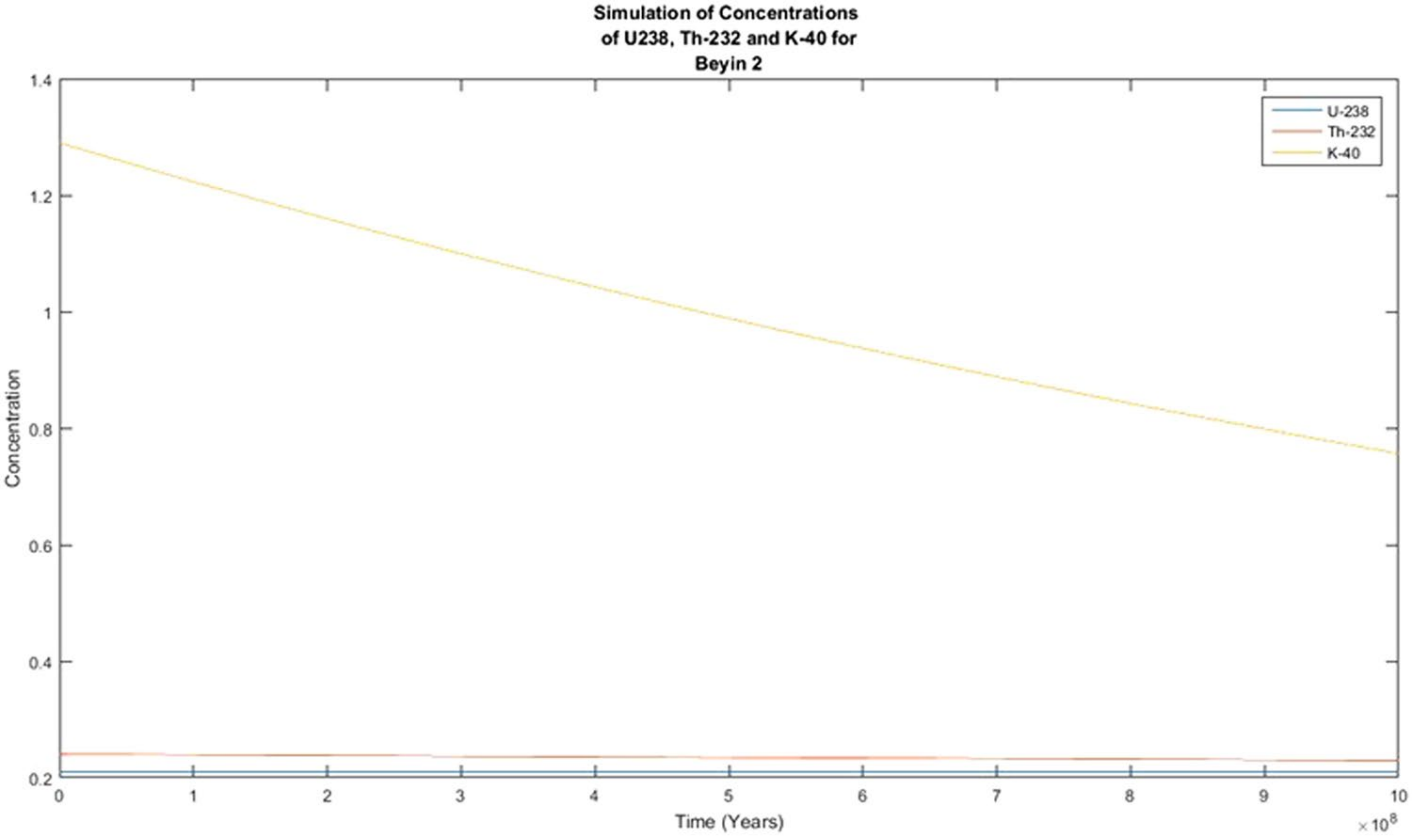
Simulation of the decay of the radionuclides ^238^U, ^232^Th and ^40^K for  ×10^8^ years.

**Figure 4:**
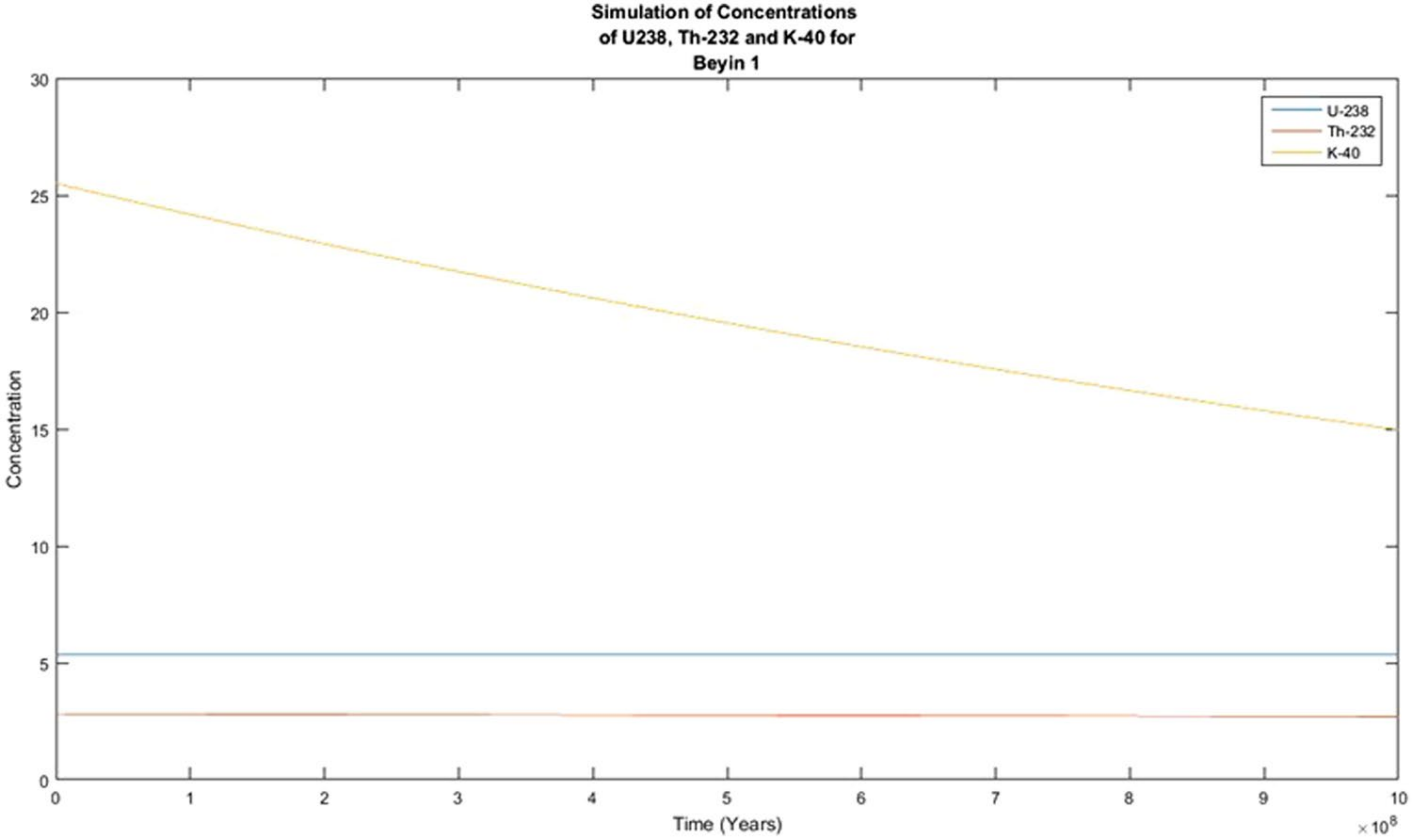
Simulation of the decay of the radionuclides ^238^U, ^232^Th and ^40^K for ×10^8^ years.

A plot of A against t was expected to give an exponential decay graph, and if the background radiation were ignored, the line would tend toward A = 0 as time goes by. This was clearly not observed in Figs 1 and 2. This is explained by the long half-lives of the radionuclides of concern. ^238^U has a half-life of 4 × 10^9^ years with that of ^232^Th being 1.4 × 10^10^ years and ^40^K being 1.25 × 10^9^ years. With these half-lives, the decay that these radionuclides will undergo in 100 years will be insignificant. As the years were increased, the decay plot of the respective radionuclides (^40^K) became more significant and tend to approach an exponential decay graph, as presented in Figs 3 and 4. This is a work in progress and will be modified with time until a full programme is developed. This programme will be used to predict levels of radionuclides in the environment, going forward. The minor decrease in the activity concentrations of ^238^U and ^232^Th will translate into minor decrease in exposure to the public expressed as the effective dose. This supposes that the impact of the radionuclides on the public will remain fairly constant for the next several years. There is the possibility of a radiological burden on people from chronic exposure to low levels of radiation, leading to the possibility of developing cancer and hereditary effects. Any future increase in the activity concentrations should be due to anthropogenic input that must be investigated to ensure the attainment of SDG 12.

## Methodology

### Study Area

The study area comprises the major communities from Axim to Newtown, which are situated along the coast bordering the Tano basin. The wells are at a water depth between 1100 and 1300 meters and at a total depth between 3400 and 4200 meters. The field covers 110 km^2^, which is about the size of 155 football pitches^31^. In geographical terms, the Jubilee field is a continuous trap with combined hydrocarbon columns in excess of 600 meters^32^. The coastal communities bordering the Tano Basin in Ghana were selected for this study due to the offshore oil and gas activities. The study area, just like any other part of Ghana, located on the equator, experiences only two seasons, the rainy (wet) and harmattan (dry) and do not experience the seasons of spring, summer, autumn, and winter. The major community is Axim. Subsistence farming is the main occupation of the people. Axim is the only coastal town with long-term climatic data within the vicinity of the Tano basin and study area. Axim experiences rainfall throughout the year. A bi-modal pattern is observed with peaks in May-June and October. The mean peak value for Axim is about 460 mm, normally in June. Axim experiences lowest rainfall of 51 mm in January. Rainfall over the sea is similar to that overland with the months of highest observed rainfall in May – June, and September – October^33^. Annual temperatures generally range from 26° C to 29 °C, with a relative humidity of 60–90% and an annual rainfall of the order of 3200 mm. The study locations have similar geologic formation composed of schists, phyllite and greywacks rocks^34^. This study is a component of wider research to establish background radioactivity for the communities from Axim to Newtown that are situated along the coast bordering the Tano basin.

### Sampling

Each community was divided into four (4) geological zones after the initial survey using a GPS. Sampling locations were selected based on accessibility to the public especially children. Soil samples were collected using a plastic trowel that was wiped clean each time prior to sampling using wipes and deionised water. Before sampling commenced, the trowel was passed through soils immediately adjacent to the sample site to remove any possible effects associated with the previous sample site^35,36^. Ten (10) soil samples were taken at 0 – 2 cm depth from each geological zone in non-coloured zip-lock plastic bags and transported to the laboratory, dried, sieved and homogenized into a composite sample. Samples were air-dried in trays for 2 weeks and then oven dried at a temperature of 105 °C for 3 to 4 minutes until the samples were well dried. Samples were milled into fine powder using Laboratory Mortar Grinder (Pulverisette-2) at the A. Chatt Chemical Laboratory of the Ghana Atomic Energy Commission. The milled samples were sieved through a 2 mm pore size mesh, homogenized and 1 kg of each composite sample weighed into 1 L Marinelli beakers. The beakers were covered and sealed with a paper tape to prevent the escape of the gaseous radionuclides in the sample. The samples were stored for at least 30 days to allow for secular equilibrium between the long-lived parent radionuclides and their short-lived progeny radionuclides in the ^238^U and ^232^Th decay series. The samples were counted on a high purity germanium (HPGe) detector for 36000 s.

### Gamma-ray spectrometry

The method of the γ-ray analysis reported in published research works^37,38^ was adopted for this study. The gamma spectrometer used for the analysis consists of an ORTEC GEM Coaxial n-type HPGe gamma-ray detector with ORTEC Multichannel Analyzer (MCA) and MAESTRO-32 evaluation software for spectrum acquisition and processing. The relative efficiency of the detector was 28.5% with an energy resolution of 1.8 keV at gamma-ray energy of 1332 keV of ^60^Co. ^238^U was determined from the average of 295.25 keV peak of ^214^Pb and 1764.5 keV peak of ^214^Bi. The gamma lines 583.19 keV and 2614.53 keV of ^208^Tl were used to determine ^232^Th and that of ^40^K was determined from the gamma line of 1460.83 keV.

The activity concentrations of ^238^U, ^232^Th and ^40^K were determined in the soil and water samples were calculated using the following analytical expression as shown in equation^37,39,40^.

2 $${{\boldsymbol{A}}}_{{\boldsymbol{sp}}}=\frac{{{\boldsymbol{N}}}_{{\boldsymbol{D}}}{{\boldsymbol{e}}}^{{{\boldsymbol{\lambda }}}_{{\boldsymbol{p}}{{\boldsymbol{T}}}_{{\boldsymbol{d}}}}}}{{\boldsymbol{p}}.{{\boldsymbol{T}}}_{{\boldsymbol{c}}}.{\boldsymbol{\eta }}.{\boldsymbol{m}}}$$

where;

N is the net counts of the radionuclide in the samples (c/s),

Td is the delay time between sampling and counting (s),

P is the gamma emission probability (gamma yield),

η is the absolute counting efficiency of the detector system,

Tc is the sample counting time (s),

m is the mass of the sample (kg),

*e* ^*λpTd*^ is the decay correction factor for delay between the time of sampling and counting, and λp is the decay constant of the parent radionuclide.

### Calculation of absorbed dose rate and annual effective dose due to radioactivity in soil samples

The activity concentrations of ^238^U in soil samples was calculated from the average energies of 295.21 and 351.92 of ^214^Pb and 609.31, 1764.49 keV of ^214^Bi. The activity concentrations of ^214^Pb and ^214^Bi in secular equilibrium with their parents were assumed to represent ^238^U activity concentration. The activity concentrations of ^232^Th were determined from the average energies of 238.63 keV of ^212^Pb, 583.19 and 2614.53 keV of ^208^Tl and 911.21 keV for ^228^Ac respectively. The activity concentrations of ^208^Tl and ^228^Ac in equilibrium with their parents were also assumed to represent the activity concentration of ^232^Th. The activity concentration of ^40^K was determined from the energy of 1460.83 keV. The absorbed dose rate from the samples was calculated from the activity concentrations of the relevant radionuclides from equation

3 $$D\,(nGy{h}^{-1})=0.0417{C}_{K}+0.462{C}_{U}+0.604{C}_{Th}$$

where

C_K_, C_U_ and C_Th_ are the activity concentrations of ^40^K, ^238^ U and ^232^Th respectively. Table 4 shows the dose conversion factors of ^40^K, ^238^U, and ^232^Th.

**Table 4:** Activity to dose rate conversion factors^9^.

Radionuclide	Dose Coefficient (nGy/h per Bq/kg)
^40^K	0.0417
^238^U	0.462
^232^Th	0.604

The annual effective dose in unit of mSv/yr was derived by converting the total absorbed dose in nGy/h and multiplying by time T of one year using the equation

4 $$E=D(nGy{h}^{-1})\times T(h{y}^{-1})\times F(\mu Sv{y}^{-1})$$

where

D is the calculated dose rate,

T is time in hours for a year given for a factor of exposure 0.20 per day throughout the year i.e.

5 $$T=0.20(24)(365+\frac{1}{4})h{y}^{-1}$$

F is the conversion factor given as 0.7 × 10^−3^ µSv/y^9,41,42^.

### Determination of radiation hazards and radiological risk assessment

#### Radium equivalent index

The radium equivalent activity, Ra_eq_, concept allows a single index or number to describe the gamma output from different mixtures of ^238^U (^226^Ra), ^232^Th, and ^40^K in a material^43^. *Ra*_*eq*_, the most frequently used indicators for the assessment of the gamma-ray radiation hazard to humans from environmental samples in Bq/kg is defined in the formula proposed by UNSCEAR^44^.

6 $$R{a}_{eq}={C}_{U}+\frac{10}{7}{C}_{Th}+\frac{10}{130}{C}_{K}$$

where C_Ra_, C_Th_, and C_K_ are the activity concentrations of ^238^U, ^232^Th, ^40^K respectively. In the definition of Ra_eq_, it is assumed that 370 Bq/kg of ^226^Ra, 259 Bq/kg of ^232^Th and 4810 Bq/kg of ^40^K produce the same gamma-ray dose rate. The above criterion only considers the external hazard due to gamma rays in building materials. The maximum recommended value of Ra_eq_ of raw building materials and products must be less than 370 Bq/kg for safe use. This means that the external gamma dose must be less than 1.5 mSv/year.

#### External hazard index (H_ex_)

Equations 3 to 5 were implemented in EXCEL spreadsheet with the concentrations of Table 2 for calculating absorbed and annual effective doses (Table 3).

The external hazard index H_ex_ was also calculated the equation

7 $${H}_{ex}=\frac{1}{370}{C}_{U}+\frac{1}{259}{C}_{Th}+\frac{1}{4810}{C}_{K}$$

#### Internal Hazard Index

The internal hazard index was calculated using the following equation

8 $${H}_{in}=\frac{1}{185}{C}_{U}+\frac{1}{259}{C}_{Th}+\frac{1}{4810}{C}_{K}$$

where C_U_, C_Th_, and C_K_ are the radioactivity concentrations in Bqkg^−1^ of ^238^U, ^232^Th, and ^40^K respectively.

#### Excess Lifetime Cancer Risk (ELCR)

Excess Lifetime Cancer Risk (ELCR) was calculated using the equation

9 $$ELCR=AEDE\times DL\times RF$$

where

AEDE is the Annual Effective Dose Equivalent

DL is the average duration of life (estimated to 70 years)

RF is the Risk Factor (Sv^-1^) i.e. fatal cancer risk per Sievert. For stochastic effects, ICRP uses RF as 0.05 for public^26,27,43,45^.

### Computational activity simulation

Newton’s forward interpolation equation is a formula designed for reconstruction of functions whose value will increase or remain constant with an independent variable (Ripa^46^). It is therefore useful for activity and dose reconstruction, against an independent variable, time. Refer to (Ripa^46^) for more information on the theory of Newton’s forward interpolation formula.

In this study, Forward Different Interpolation Method was used to reconstruct the activity concentrations of radionuclides. This was achieved by expressing the term “*e* ^−*λt*^” of the radionuclide decay equation “Α = A_0_ *e* ^−*λt*^” “nto a 4^th^ order Taylor polynomial form. The decay factor *e* ^−*λt*^ was approximated to a polynomial form by the following analysis

10 $${P}_{n}(\lambda t)={P}_{n}(z)$$

Since *P*_*n*_(*z*) = *e* ^−*λt*^ = *e* ^−*z*^; this yields the polynomial of

$$\begin{array}{rcl}{e}^{-z}={P}_{n}(z) & = & {a}_{0}+{a}_{1}(z-{z}_{0})+{a}_{2}(z-{z}_{0})(z-{z}_{1})+{a}_{3}(z-{z}_{0})(z-{z}_{1})(z-{z}_{2}+\ldots \\  &  & +\,{a}_{n}(z-{z}_{0})(z-{z}_{1})(z-{z}_{2})\ldots (z-{z}_{n-1})\end{array}$$

And the fourth order of this can be written as

$$\begin{array}{rcl}{e}^{-z}={P}_{n}(z) & = & {a}_{0}+\,{a}_{1}(z-\,{z}_{0})+\,{a}_{2}(z-{z}_{0})(z-{z}_{1})+{a}_{3}(z-{z}_{0})(z-{z}_{1})(z-{z}_{2})\\  &  & +{a}_{4}(z-{z}_{0})(z-{z}_{1})(z-{z}_{1})(z-{z}_{2})(z-{z}_{3})\end{array}$$

where

$${a}_{0}={y}_{0}={P}_{0}({z}_{0})$$

$${a}_{1}=\frac{{y}_{1}-{y}_{0}}{h}\,=\,\frac{{\rm{\Delta }}{{\boldsymbol{y}}}_{0}}{{\boldsymbol{h}}}$$

$${{\boldsymbol{a}}}_{{\bf{2}}}=\,\frac{{y}_{2}-\,2{y}_{1}+{y}_{0}}{2{h}^{2}}\,=\frac{{{\rm{\Delta }}}^{2}{y}_{0}}{2{h}^{2}}$$

$${a}_{3}=\frac{{y}_{3}-3{y}_{1}+3{y}_{1}-{y}_{0}}{2!{h}^{3}}$$

$${a}_{4}=\frac{{y}_{4}-4{y}_{3}+6{y}_{2}-4{y}_{1}+{y}_{0}}{4!{h}^{4}}$$

Such that

$${P}_{n}(z)=a{z}^{4}+b{z}^{3}+c{z}^{2}+dz+e$$

And

$$a={a}_{4}$$

$$b={a}_{3}-{a}_{4}({z}_{0}+{z}_{1}+{z}_{2}+{z}_{3}$$

$$c={a}_{2}-{a}_{3}({z}_{0}+{z}_{1}+{z}_{2})+{a}_{4}({z}_{0}{z}_{1}+{z}_{0}{z}_{2}+{z}_{0}{z}_{3}+{z}_{1}{z}_{2}+{z}_{1}{z}_{3}+{z}_{2}{z}_{3})$$

$$d={a}_{1}-{a}_{2}({z}_{0}+{z}_{1})+{a}_{3}({z}_{0}{z}_{1}+{z}_{0}{z}_{2}+{z}_{0}{z}_{3})+{a}_{4}({z}_{0}{z}_{1}{z}_{2}+{z}_{0}{z}_{1}{z}_{3}+{z}_{0}{z}_{2}{z}_{3}+{z}_{1}{z}_{2}{z}_{3})$$

$$e={a}_{0}-{a}_{1}({z}_{0})+{a}_{2}({z}_{0}{z}_{1})-{a}_{3}({z}_{0}{z}_{1}{z}_{2})+{a}_{4}({z}_{0}{z}_{1}{z}_{2}{z}_{3})$$

The 2013 version of Microsoft Excel was used to evaluate the above relations to obtain the coefficients *a, b, c, d and e* and the polynomial equals to ${e}^{-z}$ was obtained.

11 $${e}^{-z}=0.0067{z}^{2}-0.0820{z}^{3}+0.3993{z}^{2}-0.9560+1\,$$

Based on the assumption that radionuclides activity concentrations in the soils are uniform, a MATLAB R2013 script was written to simulate the decay of the radionuclides ^238^U, ^232^Th and ^40^K using their respective half-lives^47^.

### Availability of data and material

All datasets generated during this study are included in this published article.

## Conclusion

A study on natural radionuclide activity concentrations in surface soils of selected communities along the Tano basin of Ghana has been reported. The mean activity of ^238^U, ^232^Th and ^40^K were found to be 8.65 + 1.2 Bqkg^−1^, 12.5 + 1.3 Bqkg^−1^ and 214.1 + 24.3 Bqkg^−1^, respectively. Despite the fluctuation in the measurements of the activity concentrations of each natural radionuclide ^238^U, ^232^Th and ^40^K in the studied soil samples, the data are found to be normal in comparison to the worldwide standards in other countries. The average Raeq of 43.4 Bq/kg was found to be less than the recommended maximum value of 370 Bq/kg. It can be concluded that the soil may be used for the construction of buildings and may not pose any significant radiological hazards. The calculated average annual effective dose was found to be 2.51E + 01 µSv y^−1^, which is well below the permissible dose equivalent of 1 mSvy^−1^ as set by ICRP 65 and 103^26,27^. The external health hazard index for each community was found to be between 0.04 and 0.22, all below the recommended safe limit value of 1. Excess lifetime cancer risk and other radiological hazard indices were within the safe limits and therefore the exposure to soil from the studied area may not pose any immediate health hazard to the populace. This study has established baseline data for radioactivity levels in the coastal communities along the Tano oil basin in Ghana.

### Policy Recommendation

Due to the paucity of the Ghana EPA’s guideline of oil-in-water content of 29 mg/L, it is recommended that as a policy alternative that

1. As an interim measure, the Nuclear Regulatory Authority of Ghana adopts the OGP’s guidelines for the management of naturally occurring radioactive materials in the oil and gas industry. This guideline stipulates that material containing NORM above those listed in Table 5 should not be exempted from the requirements of NORM control.
  Soil shall not have a ^226^Ra contamination 0.185 Bq/g (5 pCi/g) above the background averaged over 10 m^2^ or unless risk assessment demonstrates an acceptable level risk.
  Equipment, vessels, and clothing shall be considered “NORM contaminated” if internal or external surface contamination measures double the radiation background level.

**Table 5:** Oil and gas NORM exemption level^48^.

Radionuclide	Exemption level	
	Bq/g	pCi/g
^226^Ra	1.1	30
^228^Ra	1.1	30
^210^Pb	0.2	5
^210^Po	0.2	5
^238^Pb	5.5	150
Uranium (nat)	3.0	80
2. The NRA of Ghana takes immediate steps to develop a graded approach and policy guideline (suitable and exclusive to Ghanaian conditions) based on international regulations for the management of NORMs in Ghana as has been done by ARPANSA^30^.
3. The environment and development should be properly brought together in the implementation of governmental policies for the attainment of SDG 12.

## Electronic supplementary material

Fig. S1
