# Supplementary material for: Evaluation of radionuclides and decay simulation in a terrestrial environment for health risk assessment
Source: Sci Rep. 2017 Nov 28;7:16537. doi: 10.1038/s41598-017-16659-w (PMC5705619; doi:10.1038/s41598-017-16659-w)
Supplement: Supplementary file 1 — Fig. S1 [file 41598_2017_16659_MOESM1_ESM.pdf]

Evaluation of radionuclides and decay simulation in a terrestrial environment for health risk assessment

I. N. Doyi*[1, 4], D. K. Essumang[2], S. B. Dampare[3], D. Duah[3], A. F. Ahwireng[3]

1. National Radioactive Waste Management Centre, Ghana Atomic Energy Commission, P. O. Box LG 80, Legon-Accra, Ghana
2. Department of Chemistry, School of Physical Sciences, University of Cape Coast, Cape Coast, Ghana
3. Graduate School of Nuclear & Allied Sciences, Ghana Atomic Energy Commission, P. O. Box AE 1, Kwabenya-Accra, Ghana
4. Department of Environmental Sciences, Faculty of Science and Engineering, Macquarie University, Sydney, NSW 2109, Australia

Emails: dessumang@ucc.edu.gh, sbdampare@ug.edu.gh

*Corresponding author:

E-mail: i.doyi@gaecgh.org
Tel: +233-302-401272/400310
Fax: +233-302-400807

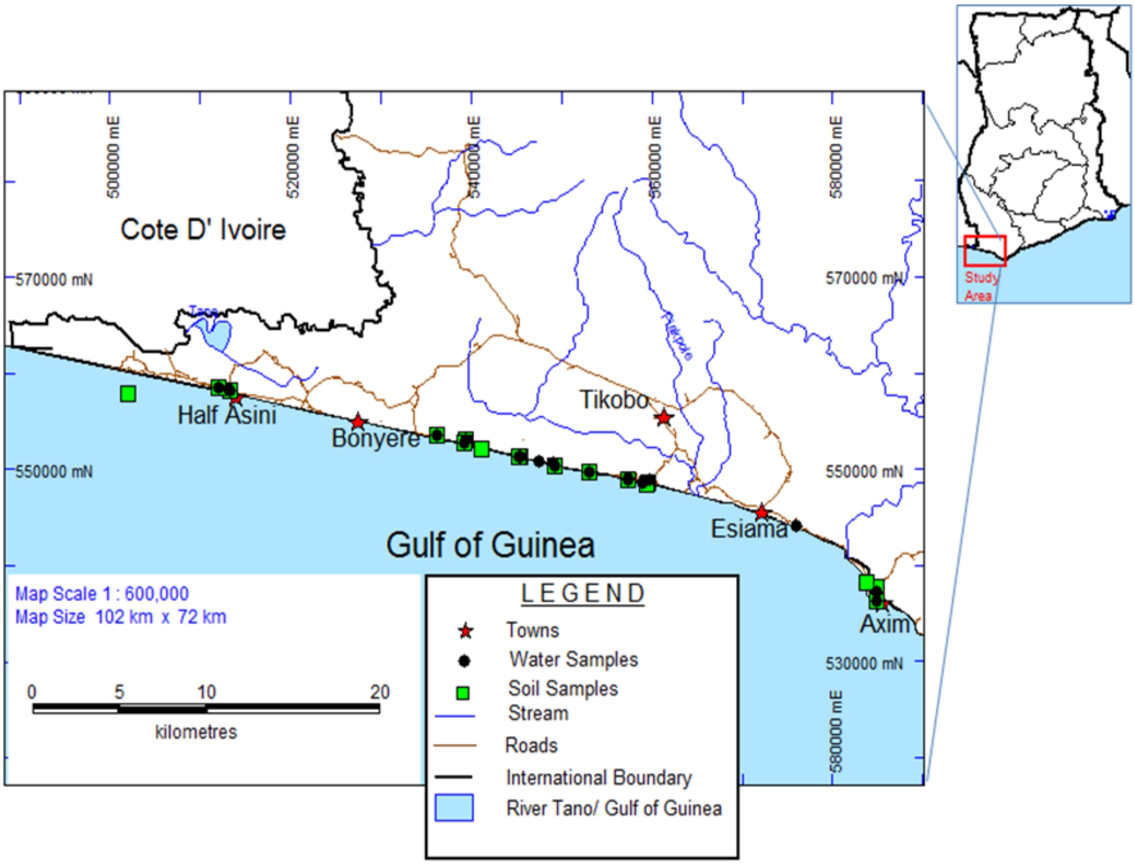

Fig. S1: Map of study area showing sampling locations (MapInfo version 8, http://mapinfo-professional.software.informer.com/8.0/).
